# Southeast Asian Foot-and-Mouth Disease Viruses in Eastern Asia

**DOI:** 10.3201/eid1803.110908

**Published:** 2012-03

**Authors:** Nick J. Knowles, JiJun He, Youjun Shang, Jemma Wadsworth, Begoña Valdazo-González, Hiroyuki Onosato, Katsuhiko Fukai, Kazuki Morioka, Kazuo Yoshida, In-Soo Cho, Su-Mi Kim, Jong-Hyeon Park, Kwang-Nyeong Lee, Geraldine Luk, Vladimir Borisov, Alexey Scherbakov, Anna Timina, Dashzeveg Bold, Tung Nguyen, David J. Paton, Jef M. Hammond, Xiangtao Liu, Donald P. King

**Affiliations:** Institute for Animal Health, Pirbright, UK (N.J. Knowles, J. Wadsworth, B. Valdazo-González, D.J. Paton, J.M. Hammond, D.P. King);; Lanzhou Veterinary Research Institute, Gansu, China (J. He, Y. Shang, X. Liu);; National Institute of Animal Health, Tokyo, Japan (H. Onosato, K. Fukai, K. Morioka, K. Yoshida);; National Veterinary Research and Quarantine Service, Gyeonggi Do, South Korea (I.-S. Cho, S.-M. Kim, J.-H. Park, K.-N. Lee);; Tai Lung Veterinary Laboratory, Hong Kong, China (G. Luk);; Federal Centre for Animal Health, Vladimir, Russia (V. Borisov, A. Scherbakov, A. Timina);; State Central Veterinary Laboratory, Ulaanbaatar, Mongolia (D. Bold);; National Centre for Veterinary Diagnostics, Hanoi, Vietnam (T. Nguyen)

**Keywords:** foot-and-mouth disease, foot-and-mouth disease viruses, viruses, epidemiology, nucleotide sequences, serotypes, viral protein 1, eastern Asia, Japan, South Korea

## Abstract

Foot-and-mouth disease (FMD) outbreaks recently affected 2 countries (Japan and South Korea) in eastern Asia that were free of FMD without vaccination. Analysis of viral protein 1 nucleotide sequences indicated that FMD serotype A and O viruses that caused these outbreaks originated in mainland Southeast Asia to which these viruses are endemic.

Foot-and-mouth disease (FMD) is a highly contagious transboundary disease that affects domesticated animals and wildlife in Africa, Asia and parts of South America. Outbreaks of FMD in these disease-endemic regions continuously threaten livestock industries in countries that are free of FMD (with or without vaccination). The causative agent, FMD virus (FMDV), is a small, nonenveloped, picornavirus (genus *Aphthovirus*) that has 7 serotypes. This virus is easily transmitted by movement of infected livestock or animal products, contaminated persons, objects, and aerosols.

FMDV serotypes O, A, and Asia 1 are endemic to countries in mainland Southeast Asia (Cambodia, Laos, peninsula Malaysia, Myanmar, Thailand, and Vietnam) where regular outbreaks of FMD have been reported (*1**,**2*). Viral protein 1 (VP1) nucleotide sequence data are widely used for phylogenetic analyses (*2**,**3*) and have been used to characterize different FMDV lineages in Southeast Asia and track transboundary movements of the virus. Studies have shown close epidemiologic links between field outbreaks in countries in Southeast Asia (*4**–**6*).

During 2009–2010, the geographic range of 2 FMDV lineages endemic to Southeast Asia (serotypes A and O) expanded into eastern Asia and caused outbreaks in 6 countries in the region. Although outbreaks of FMD caused by serotype Asia 1 have been recently reported (2005–2009) (*7*), there have been no reported outbreaks caused by serotype A in eastern Asia since 1973.

Furthermore, serotype O strains from Southeast Asia have not been detected in countries in eastern Asia since 2004 when samples were sent to the Federal Centre for Animal Health (Vladimir, Russia) from Mongolia (GenBank accession no. JQ070317) (Figure A1). In 1999–2002, extensive outbreaks caused by the PanAsia strain of serotype O were reported in Japan, South Korea, China, Taiwan, and Russia (*8**–**10*). The purpose of this study was to determine the origins of recent FMD outbreaks in eastern Asia.

## The Study

FMDVs characterized by antigen ELISA as serotype A were isolated from samples collected from FMD field outbreaks in Hubei Province, China, and in Gyeonggi-do Province, South Korea. Before FMD cases appeared in China in January 2009, serotype A infections had not occurred in that country China since 1964 (*11*), and until serotype A appeared in South Korea in January 2010, that country had been free of FMD without vaccination (for all FMDV serotypes) since 2002.

**Figure A1:**
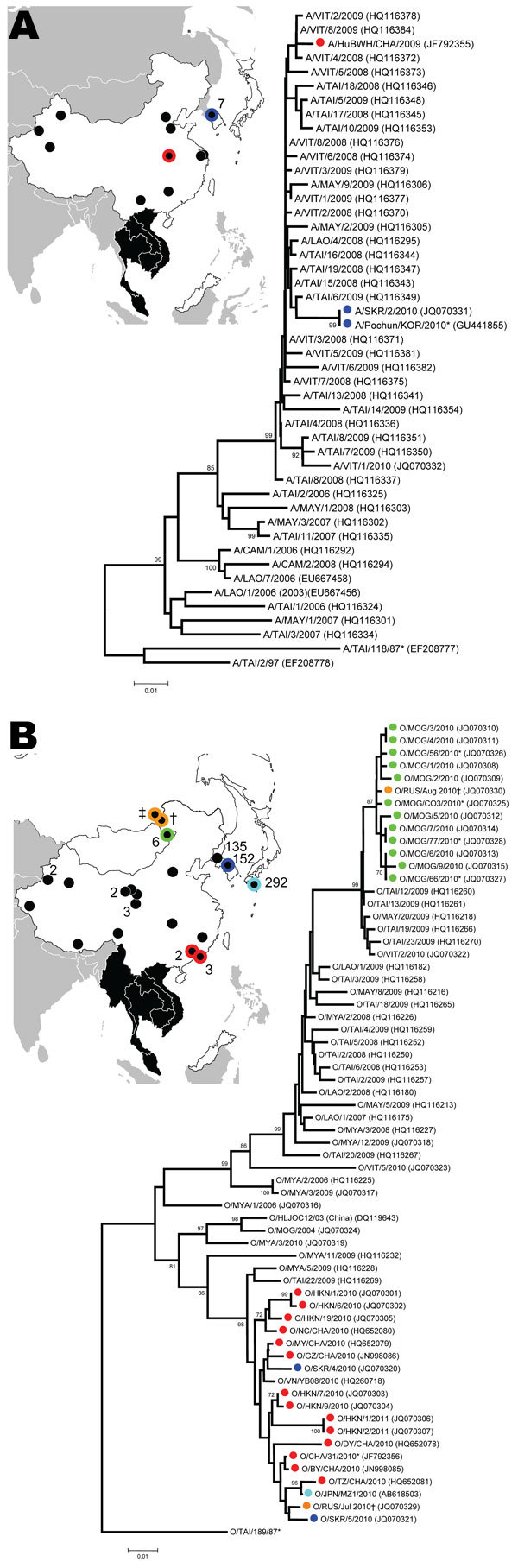
Unrooted neighbor-joining trees showing nucleotide sequence relationships between A) serotype A and B) serotype O foot-and-mouth disease (FMD) viruses from eastern Asia (red, China, including Hong Kong; dark blue, South Korea; orange, Russia; green, Mongolia; light blue, Japan) compared with closely related virus sequences from countries in mainland Southeast Asia to which FMD is endemic (black areas). Thirty-one viral protein 1 nucleotide sequences obtained during this study have been submitted to GenBank (accession nos. JQ070301–JQ070332). Additional field outbreaks reported to the World Organisation for Animal Health (OIE) for which sequence data are not available are shown as black dots (http://web.oie.int/wahis/). Percentage of replicate trees in which associated taxa clustered together in the bootstrap test (1,000 replicates) is shown next to the branches. *Reference number for sequence not assigned by the World Reference Laboratory for Foot-and-Mouth Disease. † and ‡ indicate 2 outbreaks in Russia; no sample locations were reported to OIE for O/NC/CHA/2010, O/DY/CHA/2010, O/TZ/CHA/2010, or O/MY/CHA/2010. Scale bars indicate nucleotide substitutions per site.

Phylogenetic analysis (*12*) showed that VP1 sequences obtained at Lanzhou Veterinary Research Institute (Gansu, China), the National Veterinary Research and Quarantine Service (Gyeonggi Do, South Korea), and the Institute of Animal Health (Pirbright, UK) were genetically similar to sequences obtained during 2008–2009 in Southeast Asia (Thailand and Malaysia), which belonged to the A/ASIA/Sea-97 lineage (Figure A1, panel A) (*6*). Additional FMD outbreaks caused by the same serotype that affected mainly cattle were reported in 6 provinces in China (Shanghai, Jiangsu, Guangxi, Guizhou, Shangdong, and Xinjiang) in 2009; in Beijing in January 2010 (Figure A1, panel A); and in Gyeonggi-do Province, South Korea, in January–March 2010 (6 cases).

Similar outbreaks occurred during 2010 that were caused by an FMD lineage of serotype O that is endemic to Southeast Asia (Figure A1, panel B). As of 2011, outbreaks caused by this serotype continue to occur across a wide region (Figure A1, panel B) and have affected China (including Hong Kong), South Korea, Japan, Mongolia, Russia, and North Korea.

Many FMD outbreaks (n = 292) were reported in Miyazaki Prefecture, Japan (April–June 2010). Fewer outbreaks (n = 18) were reported in China (in Guangdong, Gansu, Shaanxi, Jiangxi, and Guizhou Provinces; and in the autonomous regions of Ningxia, Xinjiang, Uyghur, and Tibet. Three outbreaks were reported in Hong Kong (February–March 2010); thirteen in South Korea (April 2010); nine, including 1 ongoing, in Mongolia (April 2010), and 2 in Russia (July and August 2010).

Japan had been free of FMD (without vaccination) since 2000, and Mongolia and Russia had not reported FMD outbreaks caused by serotype O since 2003 and 2004, respectively. These outbreaks have affected domesticated pigs, cattle, and small ruminants, and have spread to (gazelles, as represented by isolate O/MOG/9/2010) in Domod Province, Mongolia. A new increase in cases of FMD caused by serotype O has recently been reported in South Korea during December 2010 (>100 outbreaks) and in North Korea, and this serotype continues to pose a threat to livestock industries in the region.

VP1 sequences generated in the United Kingdom, China, Japan, South Korea, and Russia and those available in GenBank were analyzed by using by MEGA5 (*12*). Analysis showed that FMDVs causing serotype O outbreaks form 2 genetic clusters related to viruses within the Southeast Asia topotype (O/SEA/Mya-98 lineage), which are usually restricted to mainland Southeast Asia (Figure A1, panel B). There was >97.3% sequence relationship between sequences for FMDVs from China and those from outbreaks in Hong Kong, South Korea, and Japan.

Sequences for viruses collected in Mongolia were distinct (differing by 11.9% nt identity) and more closely related to other viruses collected during 2009 and 2010 in Southeast Asia (Thailand, Vietnam and Malaysia) (*6*). The 2 outbreaks in Russia (July and August 2010), which were caused by viruses from 2 of these sublineages, were located close to the borders with China and Mongolia and separated by ≈250 km. These outbreaks represent 2 distinct introductions of FMD into Russia.

## Conclusions

Sequence data implicate regions of mainland Southeast Asia to which FMD is endemic as the source of serotype O and A FMDVs that have caused recent outbreaks in eastern Asia. These events are not unprecedented; a previous instance of spread of FMDV from Southeast Asia into China (Yunnan Province) in 2006 involved the Asia 1 serotype. Furthermore, FMDV O/SEA topotype (Mya-98) was also detected in China in 2003 and in Mongolia in 2004. These findings provide evidence for the porous nature of borders between mainland Southeast Asia and neighboring countries and highlight the continued threat posed by FMD as a transboundary disease in the region. The extent to which viruses have spread into countries that were previously free of FMD (without vaccination) is a cause for concern.

Although VP1 sequence data can be used to characterize the viruses that are causing these outbreaks, further coordination and sharing of sequence data are now urgently required to formally identify transboundary transmission links between affected countries in the region. Complete FMDV genome sequence analyses from these field cases and additional material may provide a suitable approach to reconstruct high-resolution transmission trees and connect clusters of outbreaks (*13**,**14*).

This report describes recent incursion of FMDVs from Southeast Asia into eastern Asia. In vitro vaccine matching data (from the Institue of Animal Health) indicate that currently available vaccine strains (A/May/97and O/Manisa) should protect against representative isolates of these 2 serotypes. However, close monitoring of antigenicity and of the spread of these lineages from Southeast Asia is essential to ensure that risks for further and continued outbreaks can be mitigated.
